# Unearthing *Lactococcus lactis and Scheffersomyeces* symbionts from edible wood-boring beetle larvae as a bio-resource for industrial applications

**DOI:** 10.1186/s12866-024-03428-9

**Published:** 2024-07-30

**Authors:** Shadrack Kibet, Cynthia M. Mudalungu, Njogu M. Kimani, JohnMark O. Makwatta, James Kabii, Subramanian Sevgan, Segenet Kelemu, Chrysantus M. Tanga

**Affiliations:** 1 International Centre of Insect Physiology and Ecology (icipe), P.O Box 30772, Nairobi, 00100 Kenya; 2 Department of Physical Sciences, University of Embu, P.O Box 6, Embu, 60100 Kenya; 3 School of Chemistry and Material Science, The Technical University of Kenya, P.O Box 52428, 00200 Nairobi, Kenya

**Keywords:** Gut microbiota, Beetles, Wood-eating insects, *Lactococcus lactis*, *Scheffersomyeces*

## Abstract

**Background:**

Gut microbiota have several advantages in influencing the host nutrition, metabolism, immunity and growth. However, the understanding of the gut microbiota in key edible wood-boring beetle larvae remain largely undefined. In the present study, the characteristics of the gut microbiota of two edible wood-boring species (*Titocerus jaspideus* and *Passalus punctiger*) from two indigenous forested areas were investigated.

**Results:**

Over 50% of Amplicon Sequence Variants (ASVs) constituted of Firmicutes in *T. jaspideus*. The dominant phyla in both beetle species were Bacteroidota (4.20–19.79%) and Proteobacteria (15.10–23.90%). *Lactococcus lactis* was the most abundant and core prokaryote in the guts of *T. jaspideus*. The fungi identified in the gut of both insects belong to the phylum Obazoa (66%) and Ascomycota (> 15%). *Scheffersomyeces* sp. was the core eukaryote recorded. The diversity of gut microbiota in both insect species did not vary significantly. Most of the prokaryotic genes expressed were predominantly associated with biosynthesis and metabolism.

**Conclusion:**

Our findings demonstrated that *Lactococcus lactis* and *Scheffersomyeces* are core gut microbes of wood boring beetle larvae with desirable probiotic properties and promising use in food product fermentation for improved growth performance, gut barrier health, intestinal flora balance and immune protection for human and animals. Further studies to highlight the latest medical-based applications of *L. lactis* as live-delivery vector for the administration of therapeutics against both communicable and non-communicable diseases are warranted.

**Supplementary Information:**

The online version contains supplementary material available at 10.1186/s12866-024-03428-9.

## Background

Insects have proved to be very successful in adapting to extreme environments where food, space, and competition present significant challenges to them. To overcome these challenges, insects often get involved in vast symbiosis with other life forms such as fungi and bacteria present in their habitats. Symbiotic interactions can either be based on defensive or nutritional services provided by the symbiont to the host. Symbionts that provide nutritional services to the host majorly produce digestive enzymes aiding the degradation of plant materials that have limited insect-accessible molecules but are rich in dietary polymers such as lignin and cellulose [1]. They are also known to provide their hosts with new metabolic pathways that produce nutrients that could be lacking in their restricted diets [2]. Further, gut symbionts aid in the detoxification of noxious secondary metabolites and xenobiotic substances like terpenes [3], caffeine [4, 5], nicotine [6], and insecticides [7, 8]. For a detailed host-microbe interactions and their role in contributing to the integral aspect of the host, see Shao et al., 2024 [9]. Consequently, Class Insecta has become the most abundant on earth, both in species numbers and mass [10]. Additionally, these microbes together with their insect hosts significantly contribute to the improvement of human well-being.

Generally, as reviewed by Tanga & Ekesi [11], insects represent a promising and sustainable source of food and feed due to their efficient conversion of feed into biomass, high fecundity, minimal environmental footprint, and high nutritional profiles. Their associated microbes not only benefit their hosts but also could be tapped for human use in agriculture and industrial biotechnology [12]. Natural selection has fine-tuned the efficiency of their associated metabolites and enzymes over millions of years, offering optimized products. Unlike those from free-living microbes, symbiotic products undergo testing in eukaryotic hosts, reducing the risk of adverse effects and enhancing their suitability for human applications. Gut microbes could also be targeted in pest control strategies [13] and in the fight of vector-borne diseases [14, 15].

Microbes have proven to be vital in many systems, yet we have only scratched the surface of their roles and abundance in most insects, even those of economic and ecological importance. We lack basic knowledge about the types of insect-gut symbiotic microbes, which is crucial for understanding their functions within the insect system and possible industrial applications for the betterment of human life. This knowledge gap therefore limits our ability to explore various ways by which insects or their gut microbes can be utilised in our day-to-day lives, such as in pest control, industrial food processing, upbringing of beneficial insects, and management of infections and diseases. Xylophagous beetles in particular may have evolved intricate symbiotic system to deal with indigestible and toxic components of plants, an important strategy that could find industrial applications. In this study therefore, we sought to unravel eukaryotic and prokaryotic microbial symbionts of two wood borers, *Titocerus jaspideus* and *Passalus punctiger* by applying Next Generation Sequencing of the 16S, 18S, and ITS gene regions. The effects of phylogeny and sampling location on gut communities were investigated by analysing alpha and beta diversity metrics. Functional roles of gut bacterial community were also determined by PICRUST2 analyses.

## Methods

### Field collection of samples

**Fig. 1:**
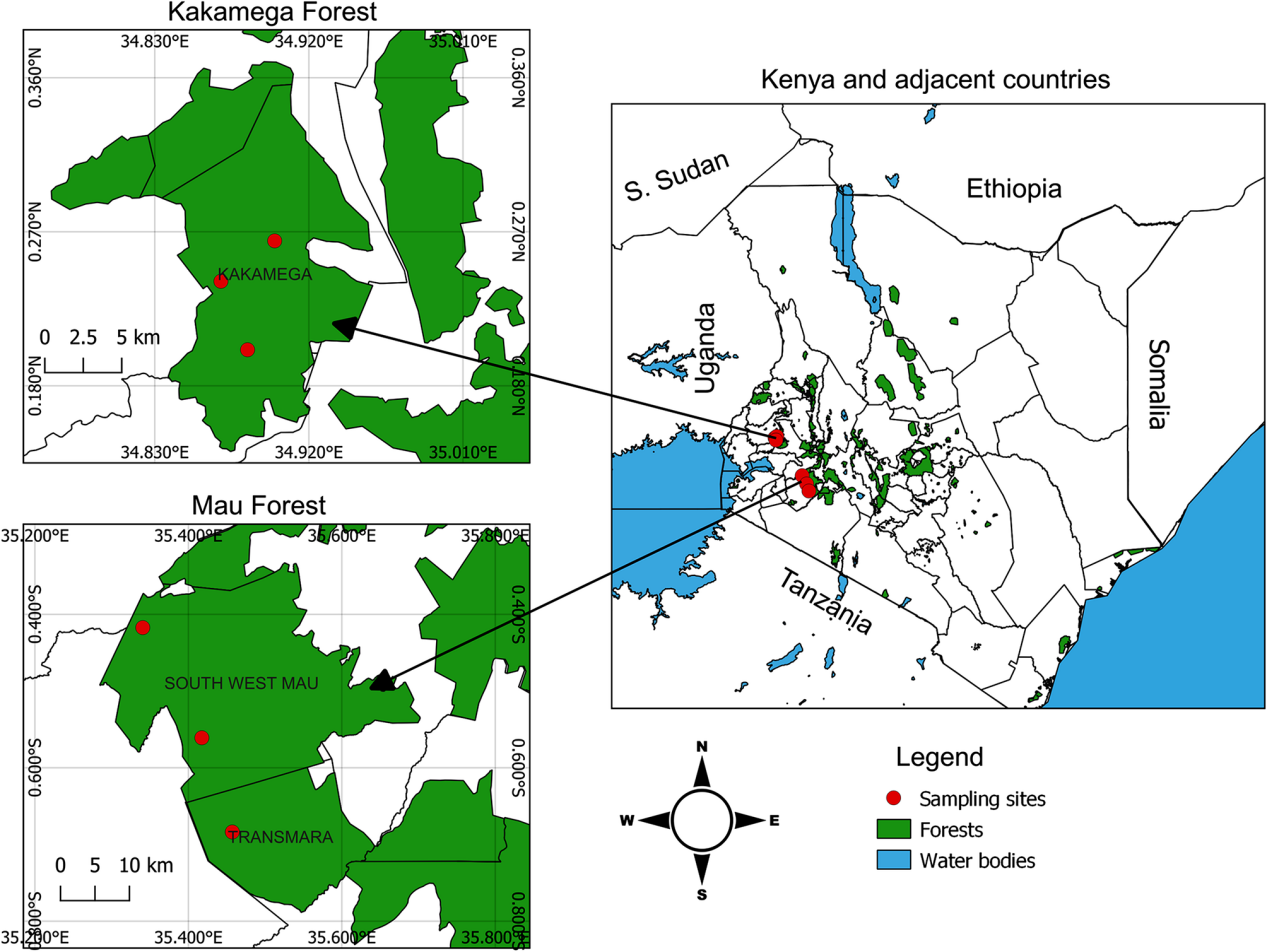
Kenyan map showing the sample collection sites

Wood burrowing beetles were sampled from Kakamega and Mau forests (Fig. 1). Sample collection was done with the authorization and guidance of the Kenya Forest Service (permit no: RESEA/1/KFS/VOL.V11-37). Specific rotting woods, known for their propensity to host beetle larvae were systematically identified and selected. The initial surveys involved a visual inspection of the forest floors to locate fallen logs displaying intermediate to advanced levels of decomposition. These logs were subjected to further screening via gentle probing, bark peeling, and visual examination of exit holes, galleries, and frass. A qualified botanist, Mr. Moses Livasia, from the Kenya Forest Service (KFS) identified the host dead woods. The larvae and recently emerged adults were carefully collected after splitting their host dead woods using an axe. In each forest, larvae were collected from three different sites, serving as replicates for the study. These specimens were preserved in cool boxes and transported to the laboratory at the International Centre of Insect Physiology and Ecology (*icipe*). To maintain their integrity, the specimens were stored at -80℃ until further experimental processes.

### Molecular identification of beetles

The frozen adults that were collected in close association with their larvae were washed using tap water and surface sterilized using 70% ethanol. They were then rinsed three times using sterile distilled water. The insect legs were detached from the body and ground in liquid nitrogen. Genomic DNA was then isolated from the ground tissue using Isolate II genomic DNA extraction kit (Bioline) while following manufacturer’s instructions. To identify the beetles, a polymerase chain reaction (PCR) was carried out with a 20 µL reaction mixture. The mixture consisted of 4 µL of HOT FIREPol^®^ Blend Master Mix, 1.0 µL each of 1.0 µM forward and reverse primers, 3 µL of DNA template, and 11 µL of nuclease-free water. The primers used were LCO1490 (5’-GGTCAACAAATCATAAAGATATTGG-3’) and HCO2198 (5’-TAAACTTCAGGGTGACCAAAAAATCA-3’) that target the cytochrome oxidase subunit 1 (CO 1), and the thermal cycling conditions were as follows: an initial denaturation at 95 ℃ for 15 min, followed by 35 cycles of denaturation at 95 ℃ for 45 s, annealing at 53 ℃ for 40 s and extension at 72 ℃ for 45 s. The final extension step was carried out at 72 ℃ for 5 min and the amplicons were held indefinitely at 4 ℃ (Proflex PCR system). PCR success was confirmed by agarose gel electrophoresis on a 2% agarose gel at 100v for 42 min and visualized using a UV trans illuminator. The amplicons were purified using ExoSap™ to remove dimers and remnant reactants. The amplicons were then sequenced in Sanger platforms at Macrogen Inc. (Amsterdam, Netherlands) and analysed by trimming, editing, and aligning using the Geneious Prime Software (Biomatter Ltd., Auckland, New Zealand) v2023.0.3. The aligned sequences were extracted and queried against the GenBank database using the Basic Local Alignment Search Tool (BLASTn) to identify similar matches. To infer the phylogeny of the beetles, a phylogenetic tree based on the nucleotide sequences of the CO1 rRNA gene was generated using MEGA software (version 11.0.13), employing the neighbour joining algorithm [16]. The analysis involved 1000 bootstraps replicates, and the Kimura two-parameter (K2P) model was utilized.

#### Gut extraction, DNA extraction, and high-throughput Illumina sequencing

Euthanized larvae samples were thawed in warm sterile water, disinfected using 70% ethanol and rinsed thrice using sterile water. The gut was then extracted by dissecting the larval cuticle and pulling out the gut using a pair of sterile forceps. The gut tissue together with the contents were then homogenized by shaking them in a tissue lyser with sterile glass beads and 0.5 mL of phosphate buffered saline (PBS, pH 7.3) for 15 min. Homogenized gut from 10 larvae per group were pooled and from which 200 µL was sampled for DNA extraction. The DNA extraction was carried out using an established CTAB protocol [17], with minor modifications. Approximately 200 µL of the samples were added to an equivalent volume of chilled CTAB buffer and a few sterile beads were incorporated. The mixture was vortexed for 1 min. The resultant solution was then topped up to 1 mL using pre-warmed CTAB and incubated at 65 ℃ for 15 min while inverting the tubes at intervals of 3 min. The end products were then centrifuged for 10 min, at 14,000 g, and the supernatant transferred to clean empty 2 mL Eppendorf tubes while the pellets were discarded. To the supernatant, equivalent volumes of phenol-chloroform-isoamyl alcohol were added, and the tubes inverted gently while ensuring that the phases mixed well. The mixture was then centrifuged at 15,000 g for 8 min, the supernatants transferred to new Eppendorf tubes and the DNA precipitated by isopropyl alcohol (70% v/v). The pellets were formed through centrifugation at 14,000 g for 15 min. The DNA pellets were washed twice using 150 µL of 70% ethanol and the final wash was carried out using 150 µL of 100% ethanol. For every wash, the wash reagent was added, shaken a bit, and centrifuged at 15,000 g for 4 min after which the supernatant was discarded. The DNA pellets were allowed to air-dry for 20 min and suspended in 70 µL of nuclease-free water. The DNA quality, as well as concentration, was determined using a Nanodrop spectrophotometer by loading about 3 µL in a 2% agarose (t = 42 min, v = 100 volts). The extracted and quantified DNA samples were stored at -80 ℃ until sequencing.

All DNA samples were sent to Macrogen for sequencing using the Illumina sequencing standard primers. For 16 S, the V3-V4 variable gene region was amplified using 341 F/805R (Forward: CCTACGGGNGGCWGCAG, Reverse: GACTACHVGGGTATCTAATCC). For ITS, ITS1-ITS2 gene region was amplified using ITS1F-ITS2 primers (Forward: CTTGGTCATTTAGAGGAAGTAA, Reverse: GCTGCGTTCTTCATCGATGC). The 18 S-V4f-V4r region of the 18 S gene on the other hand was targeted using the primer sets, forward: CCAGCAGCCGCGGTAATTCC and reverse: ACTTTCGTTCTTGATTAA.

#### Bioinformatics analysis

All the sequences (fastq) were analysed using the Divisive Amplicon Denoising Algorithm 2 (DADA2) workflow [18] implemented in R (v4.13) [19]. The pre-processing of the reads was done employing the “FilterAndTrim” function, customized with the parameters: maximum expected error rate (maxEE) of 2.5 and trimming sequence lengths at 250 and 230 for forward and reverse reads, respectively. The error rates for each sample were then computed after which the amplicon sequence variants (ASVs) together with their respective abundances were inferred using the “derepFastq” function. To merge the forward and the reverse reads, the “mergePairs” function was employed after which artefact/chimeric reads were removed using the “removeChimeraDenovo” function [20].

To assign taxonomies to the ASVs, the “assignTaxonomy” function was used, referencing the sequence reads against appropriate databases at 97% similarity thresholds. Specifically, the SILVA (version 138) reference database [21] was employed for bacterial ASV taxonomic assignments while UNITE [22] and PR2 [23] were employed for fungal and protists, respectively. The ASV counts and taxonomy tables, together with the metadata were then merged to create a phyloseq object. Following this, unwanted ASVs, such as those ascribed to chloroplasts, chloroflexi and mitochondria were removed. Reads that could not be resolved (at genus level), and therefore assigned as “NA” were filtered out. Reads from similar treatments (sampling location or species type) were pooled, and following this, taxa prevalence analysis was carried out based on absolute abundances to identify dominant taxa within the subsets. To visualize the differential prevalence of taxa, at phylum and genus levels, ggplot2 was used to plot bar plots of the top 5 phyla and top 30 genera. Those ASVs that could not feature within these thresholds (*n* = 5 and 30, for phylum and genus, respectively) were all grouped as “others” and included in the bar plots.

The alpha diversity estimates i.e. diversity, richness, and evenness were computed from a refined phyloseq object using three diversity metrics: Chao1, Shannon, and Pielou evenness, followed by either Student’s *t*-test or Mann-Whitney U test to determine if phylogeny or sampling location affected the diversity of the gut microbiota. The data normality was determined using the Shapiro-Wilk test, with a significance level set at *P* < 0.05. Prior to this, the sequence reads were rarefied by sub-sampling to achieve even depths across samples, to enable computation of alpha diversity indices. The “set.seed” function was utilized to establish a reproducible starting point for generating random numbers during the rarefaction process. Rarefaction curves were generated using R-Vegan.

Analysis of the beta diversity commenced by first creating a random tree from a non-rarefied phyloseq object. This tree was used to compute phylogenetic distances within and between groups (Bray-Curtis, weighted Unifrac distances). To test whether the groups were different with respect to centroid and dispersion, a permutation analysis of variance (PERMANOVA) with 999 permutations was performed. The beta diversity outcomes were visualized using the Principal Coordinate Analysis (PCoA) ordination plots, plotted using the ggplot2.

The core microbiome for each group of samples was determined from a non-rarefied phyloseq object merged with the random tree. In this study, the prokaryotic core microbiome was defined as those having a 75% prevalence at a detection limit of 0.001. Owing to the reduced occupancy of insect guts by eukaryotes (18 S and ITS), the eukaryotic core gut microbiota was defined as those with 30% prevalence at a detection limit of 0.001, a threshold that has been used in previous studies [13, 24]. Using the “eulerr” package in R, a Venn diagram was created to visualize the number of shared core microbiota and those unique to every treatment (sampling location or insect species).

#### Functional predictions

Phylogenetic investigation of communities by reconstruction of unobserved states (PICRUSt2) pipeline was used to predict the functional potential of microbial communities based on 16 S rRNA gene sequencing data [25]. Here, the fastq reads were imported into the PICRUSt2 and the MetaCyc pathway database was used to infer the functional gene content of the various microbial communities represented in the SILVA database of 16 S rRNA gene sequences. For visualization and data analyses, the resulting MetaCyc abundances were imported into the Statistical Analysis of Taxonomic and Functional Profiles (STAMP) software. A heat map (UPGMA, clustering threshold = 0.75) of the relatively abundant metabolic pathways was generated after which boxplots were generated to compare four of the most expressed pathways between the two insects studied.

## Results

### Sample collection and molecular identification

**Fig. 2:**
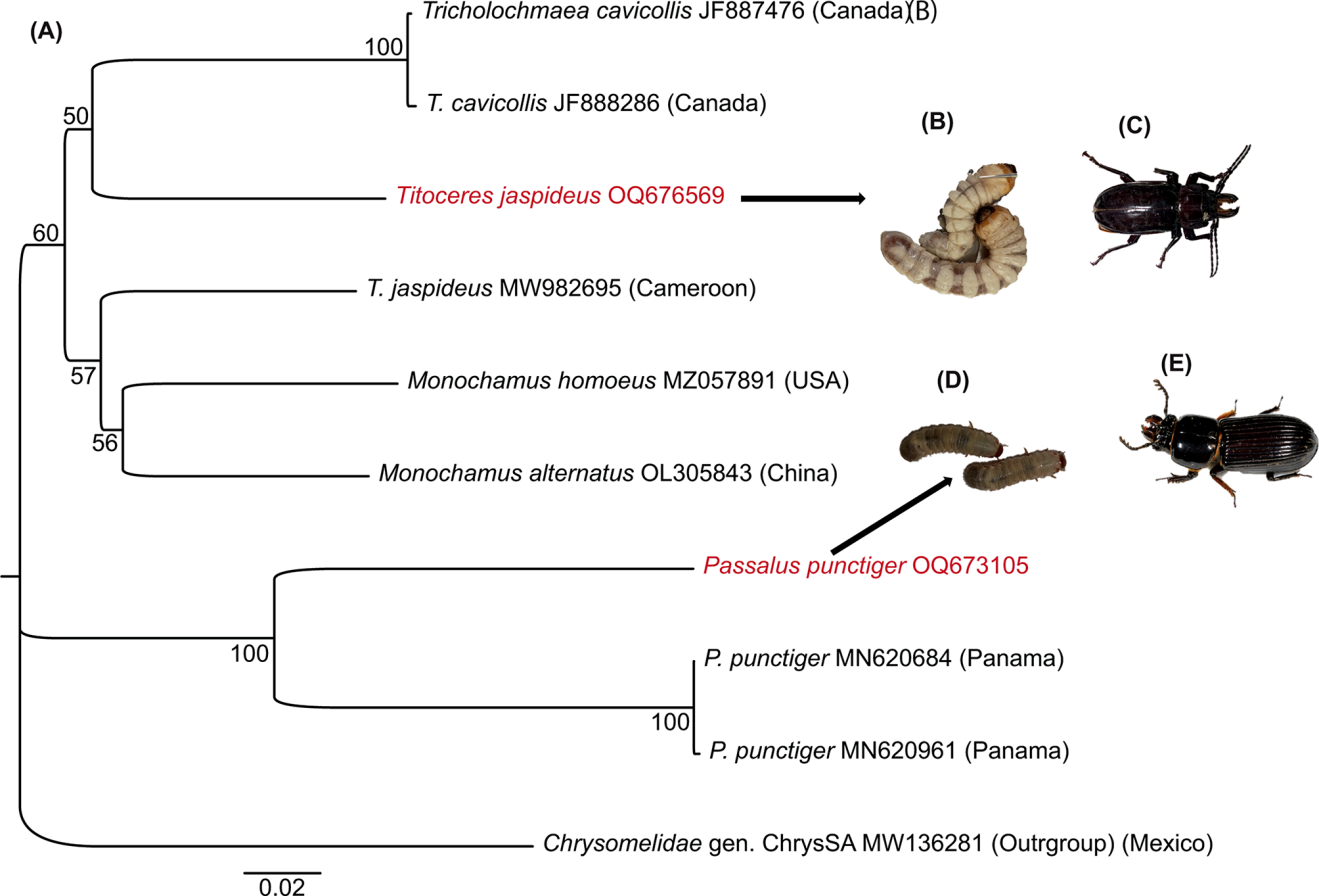
(**A**) Neighbour-joining tree based on CO1 gene sequences showing *P. punctiger* and *T. jaspideus* alongside related species. The numbers at the nodes are bootstrap values. *Chrysomelidae* (GenBank accession number MW136281) was used as an outgroup. (**B**) and (**C**) Photos of *T. jaspideus* larvae and adult, respectively. (**D**) and (**E**) Photos of *P. punctiger* larvae and adult, respectively

In both forests, two morphologically distinct wood burrowers were collected and identified from three different sites. In Kakamega forest, the two larvae were found to be dominant in decomposing logs of *Prunus africana*, *Croton megalocarpus*, *Harungana madagascariensis*, *Bridelia micrantha*, *Polyscias fulva* and *Maesopsis eminii* plants while in Mau forest, the larvae inhabited dead logs of *C. megalocarpus* and *Croton macrostachyus*. Utilizing the molecular tools, we precisely identified the two distinct beetles as *Passalus punctiger* Lepeletier, Serville (accession number OQ673105) (Fig. 2D, E) and *Titoceres jaspideus* Audinet, Serville (accession number OQ676569) (Fig. 2B, C). Phylogenetic analysis separated the two beetles into two separate clades, suggesting dissimilar phylogenies (Fig. 2A).

### Analysis of prokaryotic sequence reads

**Fig. 3:**
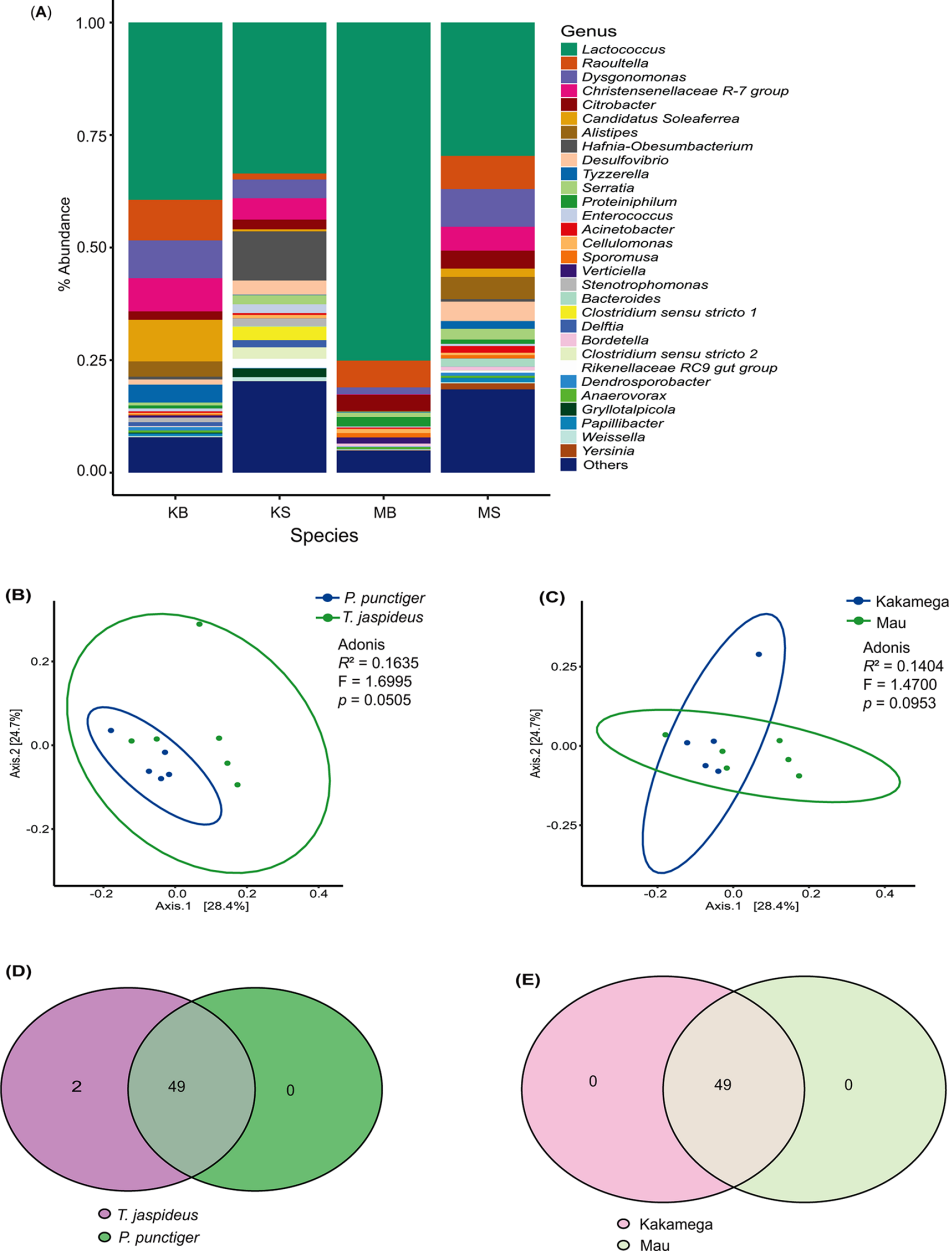
(**A**) Relative abundance of bacterial compositions at the genus level. (**B**) and (**C**) PCoA visualization plots using the Bray-Curtis dissimilarity, weighted Unifrac distances. (**D**) and (**E**) Venn diagrams showing the core bacterial ASVs. Abreviations; KB = *T. jaspideus* (Kakamega), KS = *P. punctiger* (Kakamega), MB = *T. jaspideus* (Mau), MS = *P. punctiger* (Mau)

**Fig. 4:**
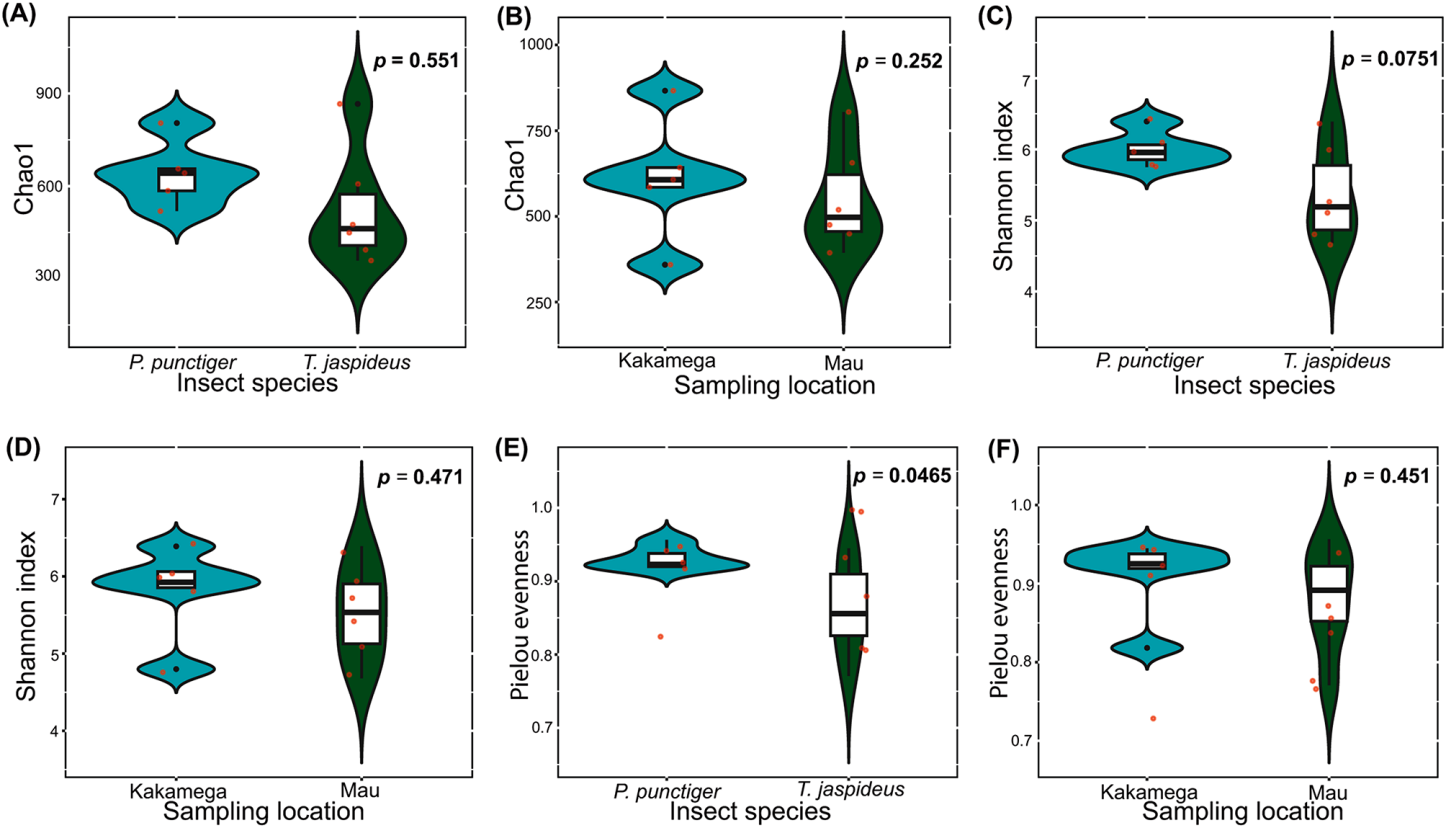
Comparison of alpha diversity metrics. (**A**) and (**B**) Chao1, (**C**) and (**D**) Shannon diversity indices, and (**E**) and (**F**) Pielou evenness values showcasing the comparison of bacterial diversity within the gut microbiota of wood borers. Points represent individual samples. Statistical significance was analysed using the Student’s *t*-tests

A summary of sequence reads obtained in every step of analysis is presented in Supplementary Table S1. The SILVA reference database initially revealed a total of 5774 ASVs. Out of these 5774, 2 chloroplasts, 5 chloroflexi and 5 archaeal ASVs were filtered out. At the Genus level, 1973 unresolved ASVs, classified as “NA” were excluded, resulting in 3789 ASVs. Eight hundred and ninety ASVs with zero taxa counts were also eliminated, resulting in 2899 ASVs across 11 samples. The rarefaction curves of all the samples analysed, (Supplementary Figure S1), plateaued, indicating adequate sampling and successful retrieval of adequate ASVs and that the sequencing depth was sufficient to access the diversity of bacterial communities [26]. Notably, Firmicutes predominated at the phylum level, representing over 50% of ASVs, particularly dominant in *T. jaspideus* compared to *P. punctiger*. Phyla Bacteroidota and Proteobacteria were also prevalent, ranging from 4.20 to 19.79% and 15.10–23.90%, respectively (Supplementary Figure S2). At the genus level, sporadically observed or low-abundance ASVs, categorized as “others,” constituted 7.90% and 4.90% of the gut microbiome of *T. jaspideus* specimens from Kakamega and Mau forests, respectively. Additionally, these ASVs accounted for 20.30% and 18.50% of the gut microbiome of *P. punctiger* specimens from the same respective forests. Among the top 30 abundant genera were *Lactococcus*,* Raultella*,* Dysgonomonas*,* Christensellenaceae R7 group*,* Citrobactor* and *Candidatus soleafera* with percent abundances ranging from 29.70 to 75.20%, 1.30–9.00%, 1.50–8.40%, 0.10 − 7.40%, 1.80–4.00% and 0.10–9.20% respectively across the samples (Fig. 3A). Notably, *Lactococcus* remained consistently more abundant in *T. jaspideus* than *P. punctiger* irrespective of the sampling site. Chao1 revealed similar bacterial richness across the two species and the two sampling locations (*P* = 0.551 and 0.252, respectively) (Fig. 4A, B). Shannon diversity showed no significant differences between the beetle species (*P* = 0.471) (Fig. 4C) or sampling locations (*P* = 0.0751) (Fig. 4D). Pielou evenness indicated a marginal significant difference between the species (*P* = 0.0456) (Fig. 4E) while sampling location had no impact (*P* = 0.451) (Fig. 4F). Similarities in the microbial community compositions between the two phylogenies and between the sampling locations were compared by PCoA based on the Bray-Curtis dissimilarity distances (weighted Unifrac). In both the PCoA plots (Fig. 3B, C), axis 1 accounted for 28.4% while axis 2 accounted for 24.7% of the data variation. The PERMANOVA analyses did not reveal discernible differences between the two insect species or the two sampling sites after 999 permutations. In the determination of the core microbiome, *T. jaspideus* was found to contain 51 core taxa while *P. punctiger* contained 49. As shown by the Venn diagram (Fig. 3D), 49 core microbiota were present in both beetle species, and *T. jaspideus* contained an additional 2 that *P. punctiger* did not (SILVA based annotation). Subsetting the samples based on the sampling locations, regardless of the phylogeny of the species, showed that there was 49 core microbiota shared by the two sampling sites as shown in (Fig. 3E). The 49 shared ASVs were all clades of *Lactococcus lactis*, belonging to the phylum Firmicutes while the two ASVs unique to *T. jaspideus* were both clades of *Raoultella ornithinolytica* of the phylum Proteobacteria.

### Analysis of eukaryotic sequence reads

The taxonomic assignments of the 18 S sequence reads utilizing the PR2 database revealed 761 taxa distributed across 10 samples. No ASVs were identified as either chloroplast, chloroflexi, mitochondrial or archaeal. At the genus level, 234 unresolved ASVs were removed, remaining with 527. The sequencing depth was adequate to access the diversity of the eukaryotes, as shown by the rarefaction curves displaying plateaus across all samples (Supplementary Figure S3). At the phylum level, Obazoa dominated accounting for 66.40–99.50% of the observed taxa. The other registered phyla included Archaeplastida and Telonemia and the SAR clade (TSAR) (Supplementary Figure S4). At the genus level, sporadically occurring ASVs accounted for 3.10% and 0.40% in *T. jaspideus*, 0.50% and 4.10% in *P. punctiger* from Kakamega and Mau, respectively.

**Fig. 5:**
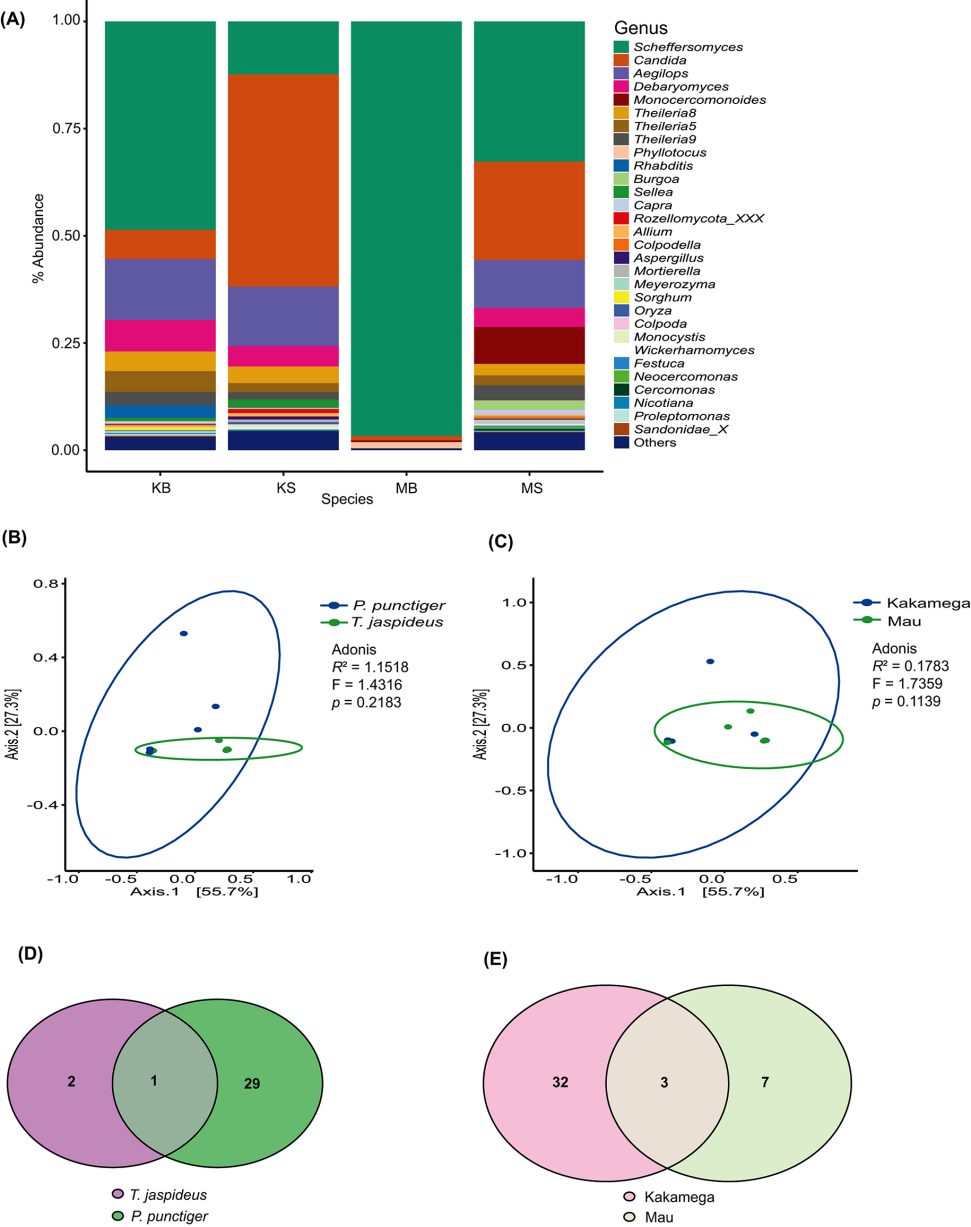
(**A**) Relative abundance of protists (18S) compositions at the genus level. (**B**) and (**C**) PCoA visualization plots using the Bray-Curtis dissimilarity, weighted Unifrac distances. (**D**) and (**E**) Venn diagrams showing shared and unshared core protist

**Fig. 6:**
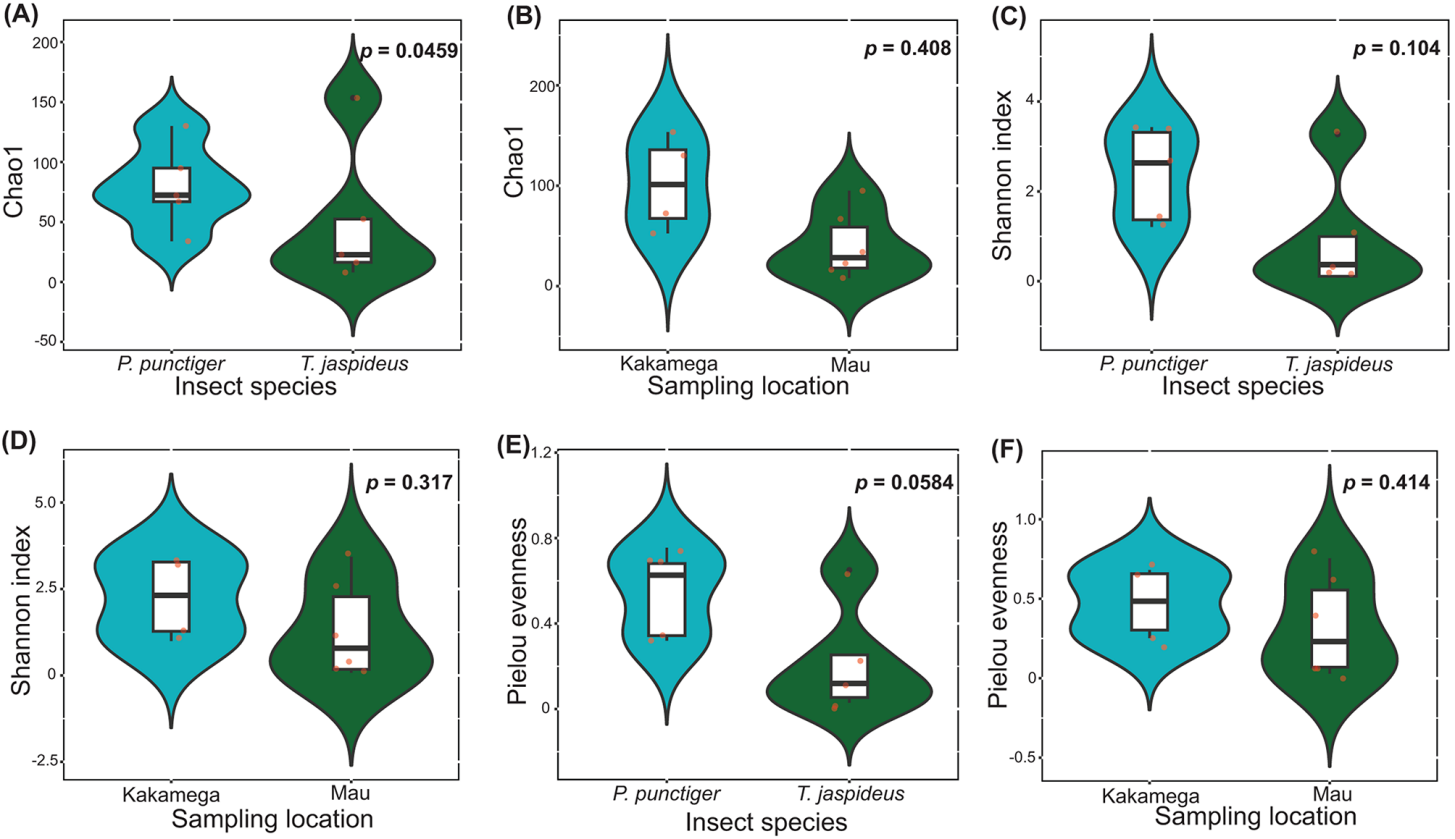
Comparison of alpha diversity metrics. (**A**) and (**B**) Chao1, (**C**) and (**D**) Shannon diversity indices, and (**E**) and (**F**) Pielou evenness values showcasing the comparison of bacterial diversity within the gut microbiota of wood borers. Points represent individual samples. Statistical significance was analysed using the Student’s *t*-tests

Among the most dominant genera *Scheffersomyces*, *Candida*, *Aegilops*, *Monocercomonoides* and *Theileria8* accounted for 12.30–96.70%, 1–49.50%, 0–14.20%, 0–8.60% and 0–4.60%, respectively across the samples (Fig. 5A). According to Chao1, there was a marginal difference in gut eukaryote richness between the two beetle species (*P* = 0.0459) (Fig. 6A) and no significant difference existed between the two sampling sites (*P* = 0.408) (Fig. 6B). Based on the diversity metric, Shannon index, neither the insect species nor sampling location affected the diversity of the gut eukaryotes (*P* = 0.104 and 0.317, respectively) (Fig. 6C, D). Similarly, Pielou evenness revealed that both sampling location and insect species did not affect the evenness of the gut eukaryotes of *T. jaspideus* and *P. punctiger* (*P* = 0.0584 and 0.414, respectively) (Fig. 6E, F).

The number of core taxa in *P. punctiger* was 23, while the number of core taxa in *T. jaspideus* was 3, out of which two were shared between the two beetles (Fig. 5D). These shared ASVs were ascribed to the genus *Scheffersomyeces*. Taking into consideration the location as a factor that would also affect the core microbiota of the two insects, Kakamega forest was associated with 34 core ASVs while Mau was associated with 11. Three ASVs, all ascribed to the genus *Scheffersomyeces*, were shared between the two locations (Fig. 5E). The beta diversity analyses based on 18 S sequence reads, by PCoA revealed that there were no significant differences between the various subset of samples (Fig. 5B, C), with axis 1 accounting for 55.7% and axis 2 accounting for 27.3% of the observed variations. Permutations of PERMANOVA (999) did not discriminate the various subsets of samples.

**Fig. 7:**
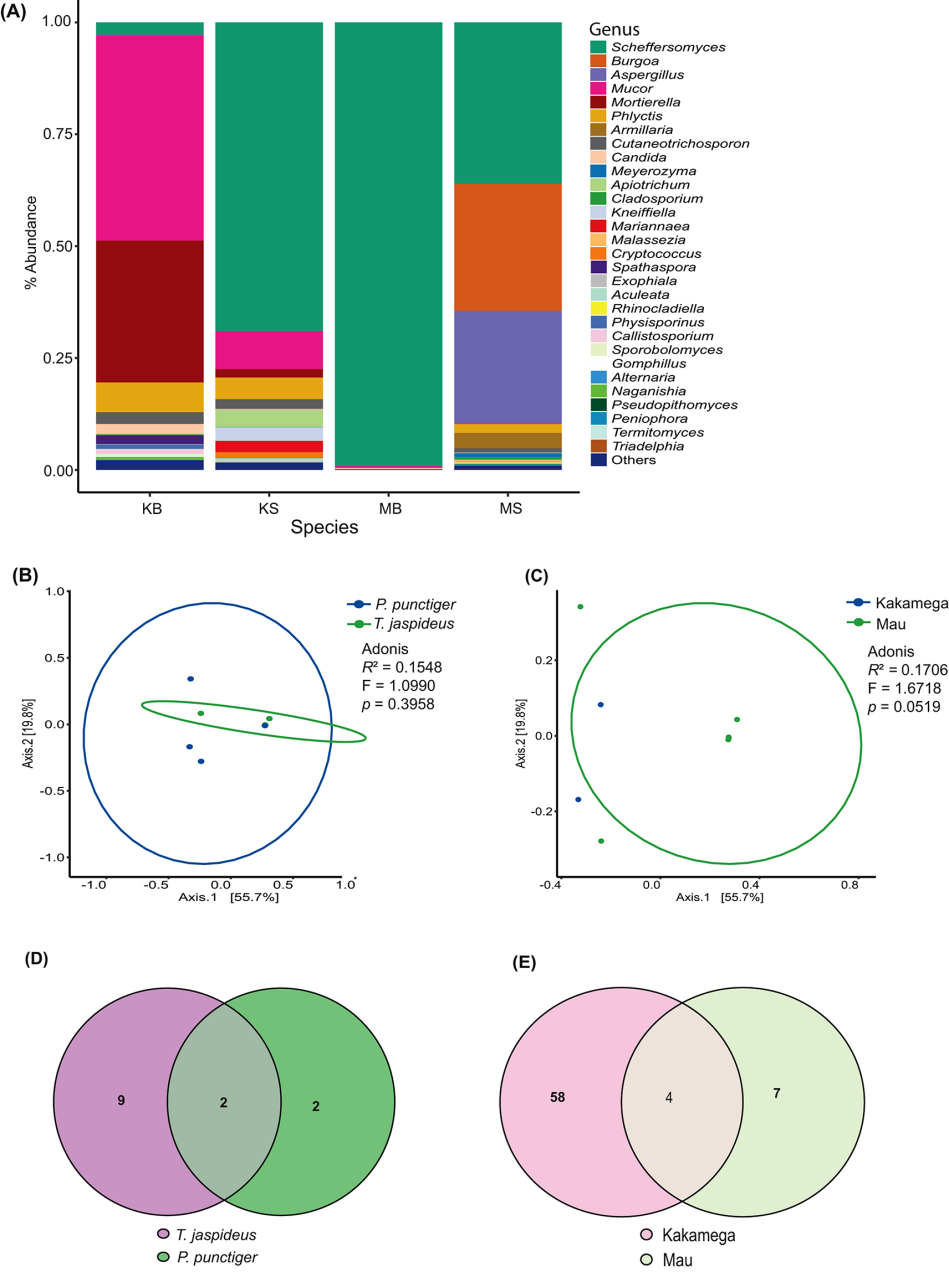
(**A**) Relative abundance of fungal (ITS) compositions at the genus level. (**B**) and (**C**) PCoA visualization plots using the Bray-Curtis dissimilarity, weighted Unifrac distances. (**D**) and (**E**) Venn diagrams showing the core fungal (ITS) ASVs. Abreviations; KS = *P. punctiger* from Kakamega, KB = *T. jaspideus* from Kakamega, MS = *P. punctiger* from Mau, MB = *T. jaspideus* from Mau

**Fig. 8:**
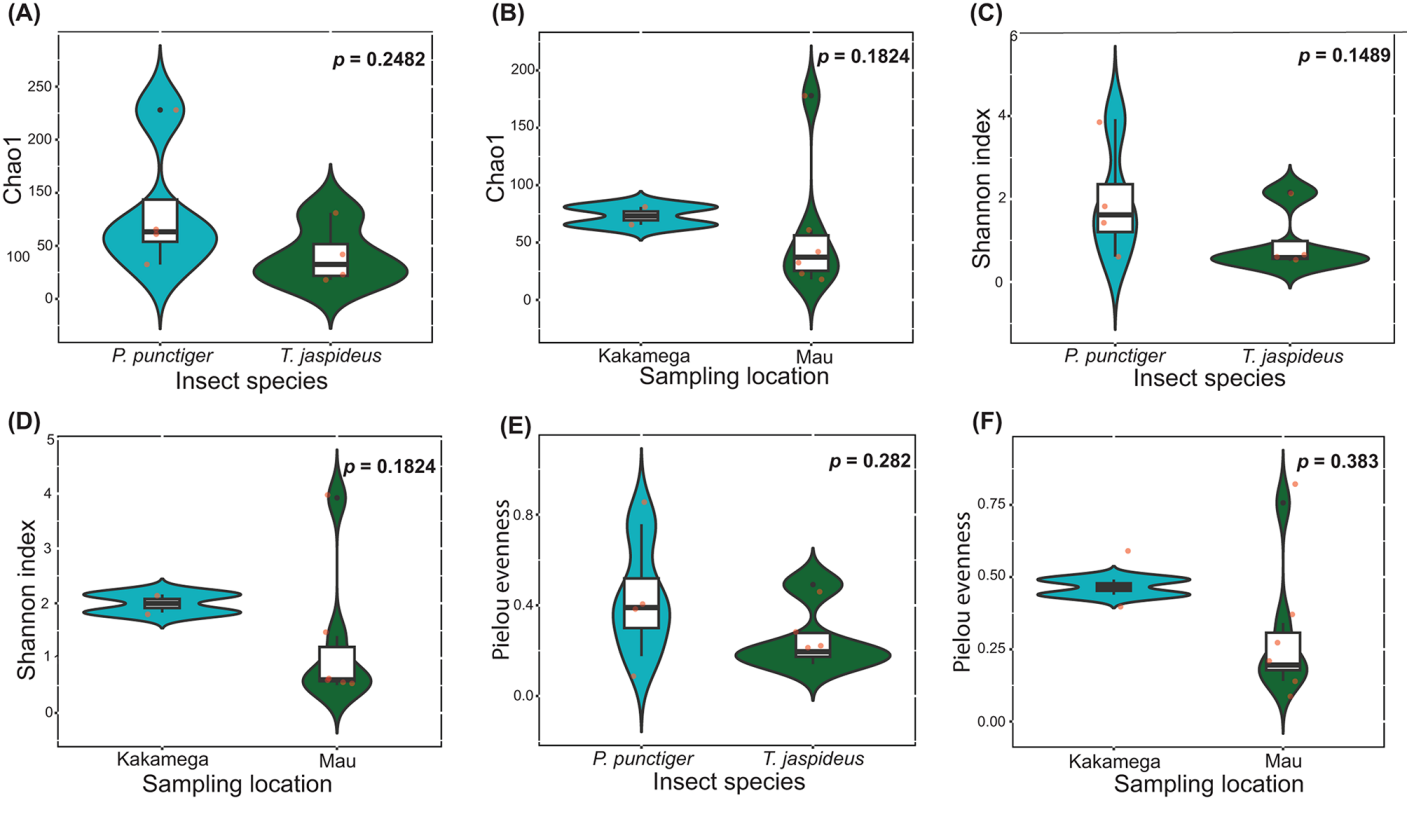
Violin plots illustrating the Chao1 diversity metrics (**A**, **B**), Shannon diversity indices (**C**, **D**), and Pielou evenness values (**E**, **F**) showcasing the comparison of fungal (ITS) diversity within the gut microbiota of wood borers, *P*-values derived from Student’s *t* test (**A**-**D**) and Man Whitney U-test (**E**, **F**)

Harnessing information from the ITS sequence reads indicated 620 ASVs distributed across 8 samples. None was classified as either chloroplast, chloroflexi, mitochondrial or archaeal ASVs. At the genus level, 216 ASVs that were classified as NAs were omitted resulting in 404 ASVs distributed across 8 samples. The rarefaction curves of the 8 samples flattened (Supplementary Figure S5), indicating adequate sampling. Among the most abundant phyla were Ascomycota, Basidiomycota, Mortierellemycota and Mucoromycota (Supplementary Figure S6). At genus level, sporadically occurring and lowly abundant genera, classified as “others” accounted for 2.20% and 1.60% for *T. jaspideus* and *P. punctiger* from Kakamega and 0.10% and 1.00% for *T. jaspideus* and *P. punctiger* from Mau forest, respectively. *Scheffersomyeces* was one of the dominant genera accounting for 99.10% and 36.00% of *T. jaspideus* and *P. punctiger*, respectively, collected from Mau forest, and 2.70% and 68.90% of *T. jaspideus* and *P. punctiger* respectively, collected from Kakamega forest (Fig. 7A). To evaluate alpha diversity metrics, a “set.seed” threshold of 30,258 reads was used, resulting in the removal of 9 ASVs upon rarefaction at even depths. Based on Chao1 both insect species and location did not affect the fungal richness of wood borers (*P* = 0.1824 and 0.2482, respectively) (Fig. 8A, B). Shannon diversity index revealed that both the phylogeny and location did not affect the diversity of the gut eukaryotes (*P* = 0.1489 and 0.1824, respectively) (Fig. 8C, D). As reflected by Pielou index, both the location and the insect species as well did not affect the evenness of the gut eukaryotes (*P* = 0.282 and 0.383, respectively) (Fig. 8E, F).

In the determination of core microbiome, *T. jaspideus* was found to have 11 core fungal ASVs while *P. punctiger* had 4. Two ASVs, identified to be strains of *Scheffersomyeces illinoinensis*, were found to be core to both species (Fig. 7D). Without taking into consideration the phylogeny, Kakamega was associated with 62 unique core ASVs while Mau had 11 (Fig. 7E). Four ASVs identified to be *S. illinoinensis* (2 ASVs), *Candida viswanathii* (1 ASV), and *Cutaneotrichosporon debeurmannianum* (1 ASV) were shared between these two sampling sites. PCoA analyses based on Bray-Curtis distances showed an overlap of various data subsets (Fig. 7B). Axes 1 and 2 accounted for 55.7% and 19.8% of the total variation observed. However, samples from Kakamega did not form an eclipse (Fig. 7C) due to failure in library construction of some of the samples from this site (Supplementary Table S1).

### Functional prediction of prokaryotic microbiota

**Fig. 9:**
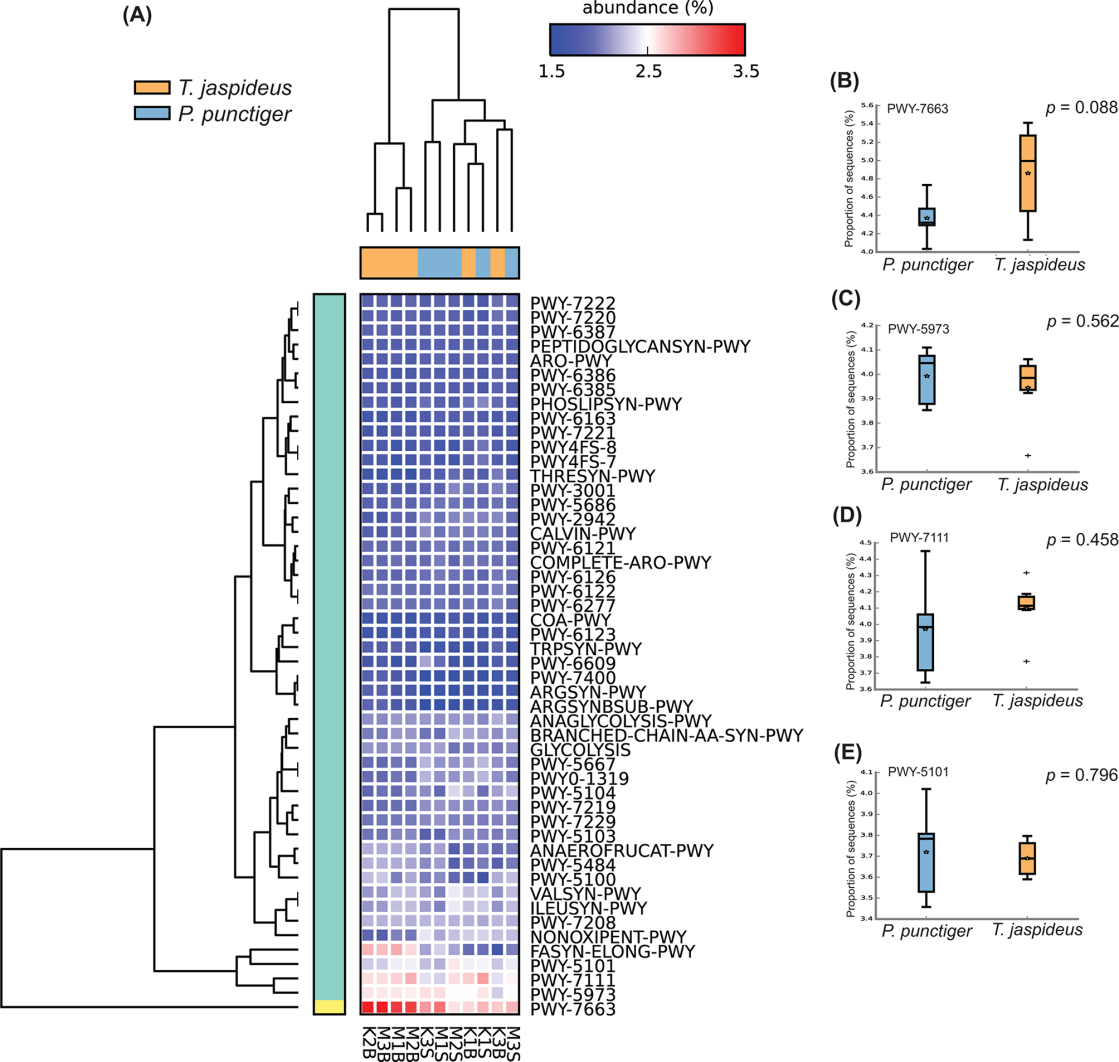
(**A**) Heatmap of the normalized relative abundances of the predicted functional categories of microbiomes associated with individual larval samples. Figures (**B**), (**C**), (**D**) and (**E**) are boxplots showing the abundances of prokaryotic gene sequences responsible for the top four expressed metacyc pathways, PWY-7663, PWY-5973, PWY-7111, and PWY-5101

In the PICRUST2 analyses, the pathways predominantly expressed were associated with metabolism and biosynthesis, specifically (PWY-7663, PWY-5973, PWY-7111, and PWY-5101) (Fig. 9A). Notably, the expression levels of these MetaCyc pathways showed no statistical significant differences between the two beetle species (Fig. 9B-E).

## Discussion

In this study, the diversity of the microbial communities that colonize the gut of two wood-eating beetle larvae, identified as *T. jaspideus* and *P. punctiger* was explored. The beetle larva *P. punctiger*, a member of the Passalidae family within the order Coleoptera, boasts adult specimens ranging from 30 to 40 mm in body length, while their larvae can extend up to 55 mm. Adults are red-brown immediately after they transition from pupae but turn to a black hue as they age [27]. On the other hand, *T. jaspideus* belongs to the long-horned beetles in the Cerambycidae family of the order Coleoptera. Adult *T. jaspideus* feature a long antenna, about half their body lengths, and can grow to about 45 mm long. Their larvae, legless white grubs, inhabit deceased logs of indigenous trees and grow to about 70 mm long [28]. These two beetle larvae share a dietary preference for dead wood despite their differing phylogenies. Hypothetically, they target this particular dietary niche to avoid triggering physiological responses in live trees, which could exert selective pressure on their microbial communities. Herein, culture-independent methods for high-throughput sequencing analysis were utilized aiming to uncover the diverse compositions of eukaryotic and prokaryotic organisms within both insect specimens. This technique has been applied widely for the determination of the gut microbiota of different insects [29–33].

This study revealed unexpected complexity in the gut communities of the two distinct beetle species that share a penchant for a limited food substrate: dead wood. Conventionally, it would be expected that species feeding on limited substrates would have a restricted gut community while those species thriving on diverse diets would harbour more intricate gut microbiota to enable them to degrade a vast variety of different nutrients [34]. The findings herein however challenge this notion and suggest that having a nutritionally complex diet such as plant lignocelluloses necessitates a diverse microbiota that assists the hosts to complete the metabolic pathways necessary to supply the demand of essential nutrients. Similar assertions have previously been made and documented in literature [35]. Notably, insects feeding on less complex substrates (sugar-rich), such as *Drosophila melanogaster* [36], hornets [37], social bees [38, 39] and the red palm weevil, *Rynchophorus ferrugineus*, [40] often have less complex microbiota due to a very specialized diet. Excess of sugar appears to reduce the complexity of the gut microbiota. The gut of *T. jaspideus* and *P. punctiger* were dominated by Firmicutes (> 50%) followed by Proteobacteria and Bacteroidota (> 4%). These results differed from that observed for invasive *Agrillus mali*, where the dominant bacteria belonged to phylum Proteobacteria, accounting for 99.7% of the assemblage [10]. Nevertheless, the results align with previous research demonstrating the prevalence of Firmicutes and Proteobacteria in various insect gut samples including wood-eating insects such as the palm weevil [40], red turpentine beetles [41], emerald ash borers [42] and other tropical wood-feeding Coleopterans [43].

The aforementioned dominant phyla confer important physiological attributes to their hosts, including impacts on nutrition, physiology, and behaviour. For instance, symbionts within the Firmicutes phylum have been shown to be pivotal in the guts of European firebugs, enabling them to exploit novel food sources and thereby expand into inaccessible ecological niches [44]. These symbionts also enhance the host’s tolerance to toxic diets [45], enhance resistance to insecticides [46, 47], and contribute to fecundity [48]. Similarly, members of Proteobacteria play vital roles in insect health, development, and survival by contributing to functions such as digestion, detoxification, immunity, pathogen resistance, and pesticide degradation [1, 49, 50]. Bacteroidetes, another important phylum, contributes to immune response modulation, a critical aspect of the host’s defense against pathogens and overall health. Considering the challenging ecological niche characterized by a complex and nutrient-deficient diet, high pathogenicity, and potential exposure to toxins within wood, the prevalence of Firmicutes, Proteobacteria, and Bacteroidetes in the gut microbiota of *T. jaspideus* and *P. punctiger* appears to be a strategic adaptation. These phyla likely play pivotal roles in enhancing these insect’s survival within their challenging habitat.

At the genus level, *Lactococcus*, specifically identified as *L. lactis* at the species level, emerged as the most prevalent microbe across all sample subsets. Employing the stated thresholds in this study (75% prevalence and 0.001 detection limits), this bacterium was found to be the core microbe in all sample subsets. Intriguingly, *Raoultella ornithinolytica* was also found to be a core microbe in samples collected from Kakamega forest. The latter has previously been reported in the gut of cycadivorous insect species and is known for its ability to convert histidine to histamine, possesses anticancer and nitrogen-fixing capabilities [51]. On the other hand, *L. lactis* is a homofermentative bacteria that ferments sugars, releasing lactic acid as the sole by-product, classifying it as a lactic acid bacterium (LAB). The substantial amounts of *L. lactis* within the gut of both *T. jaspideus* and *P. punctiger* suggests that LAB strains sourced from insects possess inherent traits conducive to surviving gastrointestinal conditions and they could potentially be offering intrinsic benefits to the hosts. For instance, administering this bacterium to *Riptortus pedestris* has been observed to elevate host survival rates, albeit no corresponding increase in the insects’ weight or size [52]. Among insect-derived LAB, symbiotic LAB found in the gut of honeybees have been studied extensively [53, 54]. Moreover, *L. lactis* has been identified in mealworms [55]. Notably, *L. lactis* holds industrial significance serving as a starter culture in fermentation of foods including vegetables, meat, wine and dairy products [56, 57]. Genetically modified forms of this bacterium have also been explored for the management of Crohn’s disease [58]. Furthermore, LAB derived from honeybees have exhibited antagonistic effects against microbial pathogens such as *Paenibacillus larvae* and *Melissococcus plutonius* [53]. With the exception of LAB being acknowledged as safe food-grade microorganisms (GRAS), it holds a promise for potential utilization as probiotics, beneficial for human health [59]. Moreover, it is beneficial in the rearing of waste-degrading insects such as black soldier fly and mealworms for food and feed [52]. Given the substantial abundance of LAB detected in the guts of *T. jaspideus* and *P. punctiger*, future steps could focus on isolating and exploring the beneficial traits of these LAB strains and their potential application, both in enhancing insect health and potentially benefiting human food industries.

Surprisingly, *Dysgonomonas*, a genus within the Bacteroidetes phylum, emerged as a strikingly represented genus (> 1%) across all samples. This genus consists of fermentative anaerobes that produce acids and no gas and were first isolated from an infected human gall bladder [60]. While classified as an opportunistic human pathogen, their predominant habitats remain largely elusive. Recent studies have however found that this genus is widely distributed in terrestrial environments and is particularly enriched in insect systems [61]. Its members have been detected in microbial fuel cells (MFC) anode biofilms [62] and in the gut of house flies [63]. The detection of *Dysgonomonas* in wood-boring insects warrants further inquiry to determine their actual putative role. Another notable genus associated with the two beetles was *Enterococcus* belonging to the family Enterococaceae, a genus that has previously been associated with ability to degrade alkaloids, which may play a putative role in the polyphagous hosts’ tolerance to toxic plants [45]. This genus has thus been widely detected in plants-eating insects such as *Hyles euphorbiae* and *Brithys crini* [45], *Spodoptera litura* [64], *Bombyx mori* [65]. Additionally, it has been demonstrated that the dominance of *Enterococcus* in the gut of *Galleria mellonella* larvae plays an immune-modulatory role in protecting the host against pathogenic bacteria and fungi [66]. Such capabilities can be very important in allowing insects to overcome plant defense mechanisms and adapt to a complex digestive and pathogenic environment.

Other dominant taxa detected across the samples were *Citrobacter* and the R-7 group of the *Christensenellacae*. The precise role of the *Christensenellaceae* R-7 group within the insect gut microbiomes remains incompletely elucidated, however, this group has been associated with amino acids and lipids metabolism in alpine musk deer [67]. Nonetheless, congruent to our findings, a recent study identified this taxon in the larval paunch of *Cotinis nitida* [68]. The presence of this taxon in the human gut is associated with improved metabolic health [69, 70]. *Citrobacter* on the other hand has been found in the gut of several insects including weevils, termites, flies, and moths [71]. This group is crucial to its host as it contributes to nitrogen recycling, insecticide resistance, and aiding in digestion such as in cellulose degradation [50, 71, 72].

Analysis of protists and fungal members of the gut was faced with significant impediments. Four samples of ITS and two of 18 S (Supplementary Information Table S1) failed to progress past the library construction phase despite repeated attempts. However, absence of fungi has been observed in another wood-borer, the invasive *Agrilus mali*, even though this absence was attributed to selective inhibition of fungi by gut bacteria [73]. Nevertheless, our study managed to detect phyla Obazoa, Archaeplastida, and TSAR eukaryotic super-group following amplification and sequencing of the 18 S gene region. The ITS gene region on the other hand led to the detection of phyla Ascomycota, Basidiomycota, Mortierellomycota and Mucoromycota. The dominant fungal phyla (Ascomycota and Basidiomycota) observed in *T. jaspideus* and *P. punctiger* are consistent with previous observations of their high abundance in Cerambycidae, Passalidae, Elateridae, Hemiptera, and Lepidopterans [74–77]. Generally, these phyla are known to contribute to various functions such as immune response, potential biocontrol, and the provision of necessary digestive enzymes [76]. Consequently, they play a role in expanding the host’s dietary options and habits. Though the habitat of most Mortierellomycota is known to be soil, we were able to detect this phylum from insect samples collected from the Kakamega forest only. This phylum has also been detected in other insects such as *Agriophara rhombata* [78]. Their presence in samples collected from one locality hypothetically infers that this group is site specific and possibly originated from the rich microfloral soil found in Kakamega forest.

Subsequently, the most predominant genus was *Scheffersomyeces*, also identified as a shared core microbe, based on both ITS and 18 S annotations. Its presence and high abundance has been widely documented in various wood-eating insects [79–81]. Members of this genus can ferment xylose, the second most abundant monosaccharide in woody plant biomass, producing L-lactic acid, ethanol, and other by-products [82]. The presence of *Scheffersomyeces* in the gut of *T. jaspideus* and *P. punctiger* is of interest due to its role in degradation of wood and its potential industrial applications in the lignocellulosic biofuel industry. Future studies ought to be guided to isolate and characterise these strains and determine their degradative capabilities in vitro.

Examining the effects of sampling locations and phylogeny on the diversity indices revealed no significant differences for alpha diversity (*P* > 0.05, Student’s t-test or Mann-Whitney U test) and beta-diversity (*P* > 0.05, PERMANOVA in 1000 permutations) for both prokaryotic and eukaryotic microbes. Furthermore, PCoA analysis neither discriminated the samples based on the sampling locations nor phylogeny. Absence of notable difference in the diversity indices indicates that both phylogeny and the sampling location did not influence the distribution and the load of the gut microbial communities of the two larvae. These findings suggest that factors beyond phylogeny or specific habitat might predominantly shape and regulate the gut microbial communities in these beetles. The similarity in diet fed on by these two larvae and the lack of significant differences observed in both alpha and beta diversities could suggest that the diet of the organism may primarily influence the composition of gut microbial communities. This hypothesis aligns with previous studies indicating that even within a species, diet dictates the shape of the microbiota of an individual [83, 84].

The prediction of functional roles of gut microbiota revealed that the most abundant pathways were associated with biosynthesis and metabolism. The pathway PWY-7663, that was strongly expressed is a gondoate biosynthetic pathway, indicating probable importance in the gut of these two insects. The detection of PWY-7111, a pyruvate to isobutanol biosynthetic pathway, is particularly intriguing. Being an engineered pathway, its presence and high abundance across the samples in this study suggest inclusion in the list of naturally occurring pathways. Enzymes that catalyse this biosynthesis have been isolated from several organisms, including *L. lactis* [85], the core prokaryote in our analysis, underscoring the mosaic nature of microbial functional capabilities within gut microbiomes.

## Conclusions

This study reports for the first time the gut microbiota of wood burrowing beetles; *Titocerus jaspideus* and *Passalus punctiger*. Intestinal guts of these beetles constitute a rich microbial habitat, challenging the notion that species feeding on a restricted diet have less complex gut microbiota. Firmicutes, Proteobacteria, Ascomycota, Basidiomycota, Obasoa and TSAR dominated the guts of these beetles. *Lactococcus lactis* and *Scheffersomyeces* sp, fermentative microbes with potential for industrial application in food fermentation, were identified as the core microbes. These microbes could be playing pivotal roles in nutrition, physiology, and behaviour. Despite differences in sampling locations and phylogeny, no significant impact on microbial diversity was observed, suggesting that diet might be a primary influencer of gut microbial composition. Predicted functional roles indicated a strong emphasis on biosynthesis and metabolism, with intriguing pathways such as gondoate and pyruvate to isobutanol biosynthesis. The findings underscore the intricate relationships between wood-eating beetles, their gut microbiota, and the implications of identified microbes in various industries. It is essential to note that while our study provides valuable insights into the gut microbiota composition of wood-burrowing beetles, the DNA extracted from the gut represents a snapshot of the microbial community, which may include inactive or dead microbes. Future studies utilizing RNA fraction analysis could offer deeper insights into the active microbial populations.

### Electronic supplementary material

Below is the link to the electronic supplementary material.

Supplementary Material 1

## Data Availability

The datasets generated and/or analysed during the current study are available in the NCBI repository: 16S accession numbers- SRR28384120 – SRR28384130, 18S accession numbers- SRR28384267 – SRR28384276 and ITS accession numbers- SRR28384291 – SRR28384298.
