# Supplementary material for: Unearthing Lactococcus lactis and Scheffersomyeces symbionts from edible wood-boring beetle larvae as a bio-resource for industrial applications
Source: BMC Microbiol. 2024 Jul 30;24:282. doi: 10.1186/s12866-024-03428-9 (PMC11290184; doi:10.1186/s12866-024-03428-9)
Supplement: Supplementary file 1 — Supplementary Material 1 [file 12866_2024_3428_MOESM1_ESM.docx]

**Unearthing *Lactococcus lactis* and *Scheffersomyeces* symbionts from edible wood-boring beetle larvae as a bio-resource for industrial applications**

**Kibet Shadrack^1,2^, Cynthia M. Mudalungu^1,3*^, Njogu M. Kimani^2^, JohnMark O. Makwatta^1^, James Kabii^1^, Segenet Kelemu^1^ and Chrysantus M. Tanga^1*^**

^1^International Centre of Insect Physiology and Ecology (*icipe*), P.O Box 30772 – 00100, Nairobi, Kenya

^2^Department of Physical Sciences, University of Embu, P.O Box 6 – 60100, Embu, Kenya

^3^School of Chemistry and Material Science, The Technical University of Kenya, P.O Box 52428-00200, Nairobi, Kenya

**Correspondence:** [cmudalungu@icipe.org](mailto:cmudalungu@icipe.org)

[ctanga@icipe.org](mailto:ctanga@icipe.org)

Supplementary Table S1. Summary reads

| **Gene region** | **Individual** | **Sampling site** | **Input reads** | **Filtered reads** | **Denoised reads** | **Merged reads** | **Tabled reads** | **Non-chimeric reads** | **Assigned reads** | **Seed set** | **Chao1** | **Pielou evenness** | **Shannon** | **Phyla** |
| --- | --- | --- | --- | --- | --- | --- | --- | --- | --- | --- | --- | --- | --- | --- |
| Bacterial 16S rRNA gene | K1B | Kakamega forest | 61883 | 58733 | 56285 | 49841 | 49841 | 27347 | 21844 | 15930 | 866.4516 | 0.9448 | 6.3895 | 9 |
| Bacterial 16S rRNA gene | K2B | Kakamega forest | 50008 | 47749 | 47256 | 44837 | 44837 | 22976 | 20987 | 15930 | 359 | 0.8182 | 4.7998 |  |
| Bacterial 16S rRNA gene | K3B | Kakamega forest | 71769 | 68592 | 66548 | 58696 | 58696 | 28867 | 24459 | 15930 | 607 | 0.9252 | 5.9248 |  |
| Bacterial 16S rRNA gene | K1S | Kakamega forest | 56695 | 52334 | 49654 | 43351 | 43351 | 20599 | 15930 | 15930 | 642.3333 | 0.9379 | 6.0631 | 12 |
| Bacterial 16S rRNA gene | K2S | Kakamega forest | NP |  |  |  |  |  |  |  |  |  |  |  |
| Bacterial 16S rRNA gene | K3S | Kakamega forest | 94969 | 82265 | 80004 | 73610 | 73610 | 36207 | 27360 | 15930 | 584.8 | 0.9193 | 5.8542 |  |
| Bacterial 16S rRNA gene | M1B | Mau forest | 95437 | 83571 | 82380 | 78374 | 78374 | 33121 | 22250 | 15930 | 393.7692 | 0.8483 | 5.0635 | 7 |
| Bacterial 16S rRNA gene | M2B | Mau forest | 70679 | 67447 | 65433 | 58373 | 58373 | 26225 | 21235 | 15930 | 475.12 | 0.8633 | 5.3189 |  |
| Bacterial 16S rRNA gene | M3B | Mau forest | 96270 | 83738 | 81924 | 74513 | 74513 | 45384 | 42126 | 15930 | 449.3659 | 0.7707 | 4.6804 |  |
| Bacterial 16S rRNA gene | M1S | Mau forest | 78128 | 65372 | 62512 | 51198 | 51198 | 30672 | 25086 | 15930 | 519.1429 | 0.923 | 5.7506 | 9 |
| Bacterial 16S rRNA gene | M2S | Mau forest | 68794 | 65533 | 61736 | 47825 | 47825 | 25690 | 19707 | 15930 | 804.5263 | 0.9564 | 6.3922 |  |
| Bacterial 16S rRNA gene | M3S | Mau forest | 80335 | 76948 | 71185 | 52189 | 52189 | 30412 | 22539 | 15930 | 656.1429 | 0.9199 | 5.9556 |  |
| Fungal ITS gene region | K1B | Kakamega forest | NP |  |  |  |  |  |  |  |  |  |  | 4 |
| Fungal ITS gene region | K2B | Kakamega forest | NP |  |  |  |  |  |  |  |  |  |  |  |
| Fungal ITS gene region | K3B | Kakamega forest | 87788 | 57715 | 57515 | 56476 | 56476 | 56140 | 30258 | 30258 | 81 | 0.4915 | 2.16 |  |
| Fungal ITS gene region | K1S | Kakamega forest | NP |  |  |  |  |  |  |  |  |  |  | 4 |
| Fungal ITS gene region | K2S | Kakamega forest | NP |  |  |  |  |  |  |  |  |  |  |  |
| Fungal ITS gene region | K3S | Kakamega forest | 125854 | 105819 | 105604 | 104799 | 104799 | 99214 | 45830 | 30258 | 65.5 | 0.4392 | 1.8332 |  |
| Fungal ITS gene region | M1B | Mau forest | 112829 | 104845 | 104699 | 104005 | 104005 | 103367 | 103315 | 30258 | 18 | 0.207 | 0.5983 | 3 |
| Fungal ITS gene region | M2B | Mau forest | 94893 | 84718 | 84648 | 84490 | 84490 | 84348 | 84320 | 30258 | 23 | 0.1837 | 0.5761 |  |
| Fungal ITS gene region | M3B | Mau forest | 148472 | 132417 | 132289 | 131949 | 131949 | 131860 | 130902 | 30258 | 42 | 0.1408 | 0.5083 |  |
| Fungal ITS gene region | M1S | Mau forest | 134714 | 108284 | 108084 | 105944 | 105944 | 105092 | 101783 | 30258 | 61 | 0.3411 | 1.402 | 3 |
| Fungal ITS gene region | M2S | Mau forest | 104527 | 77705 | 77106 | 68447 | 68447 | 67217 | 66490 | 30258 | 178 | 0.7572 | 3.9236 |  |
| Fungal ITS gene region | M3S | Mau forest | 107029 | 100637 | 100509 | 99748 | 99748 | 99413 | 75029 | 30258 | 32.5 | 0.1761 | 0.6104 |  |
| Fungal 18S gene region | K1B | Kakamega forest | 95572 | 54455 | 53910 | 52655 | 52655 | 52135 | 48737 | 17967 | 153.5 | 0.6506 | 3.2683 | 4 |
| Fungal 18S gene region | K2B | Kakamega forest | NP |  |  |  |  |  |  |  |  |  |  |  |
| Fungal 18S gene region | K3B | Kakamega forest | 81487 | 79268 | 79240 | 77245 | 77245 | 77191 | 74758 | 17967 | 52.5 | 0.253 | 0.9949 |  |
| Fungal 18S gene region | K1S | Kakamega forest | 59553 | 37453 | 37317 | 36153 | 36153 | 35455 | 32454 | 17967 | 130 | 0.6805 | 3.3124 | 4 |
| Fungal 18S gene region | K2S | Kakamega forest | NP |  |  |  |  |  |  |  |  |  |  |  |
| Fungal 18S gene region | K3S | Kakamega forest | 83756 | 81113 | 80965 | 80727 | 80727 | 80584 | 69015 | 17967 | 72.33333 | 0.3198 | 1.3675 |  |
| Fungal 18S gene region | M1B | Mau forest | 95739 | 91762 | 91724 | 91586 | 91586 | 91281 | 90814 | 17967 | 16.33333 | 0.0287 | 0.0797 | 2 |
| Fungal 18S gene region | M2B | Mau forest | 119261 | 114513 | 114440 | 114270 | 114270 | 113788 | 113571 | 17967 | 8 | 0.0533 | 0.1108 |  |
| Fungal 18S gene region | M3B | Mau forest | 119837 | 116817 | 116763 | 116723 | 116723 | 116671 | 105393 | 17967 | 22.75 | 0.1195 | 0.3694 |  |
| Fungal 18S gene region | M1S | Mau forest | 36074 | 33549 | 33488 | 22473 | 22473 | 22447 | 17967 | 17967 | 67 | 0.626 | 2.6322 | 5 |
| Fungal 18S gene region | M2S | Mau forest | 56633 | 48702 | 48605 | 46915 | 46915 | 46669 | 42909 | 17967 | 95 | 0.7549 | 3.4299 |  |
| Fungal 18S gene region | M3S | Mau forest | 88973 | 85893 | 85859 | 85530 | 85530 | 85099 | 81243 | 17967 | 34 | 0.3432 | 1.2102 |  |


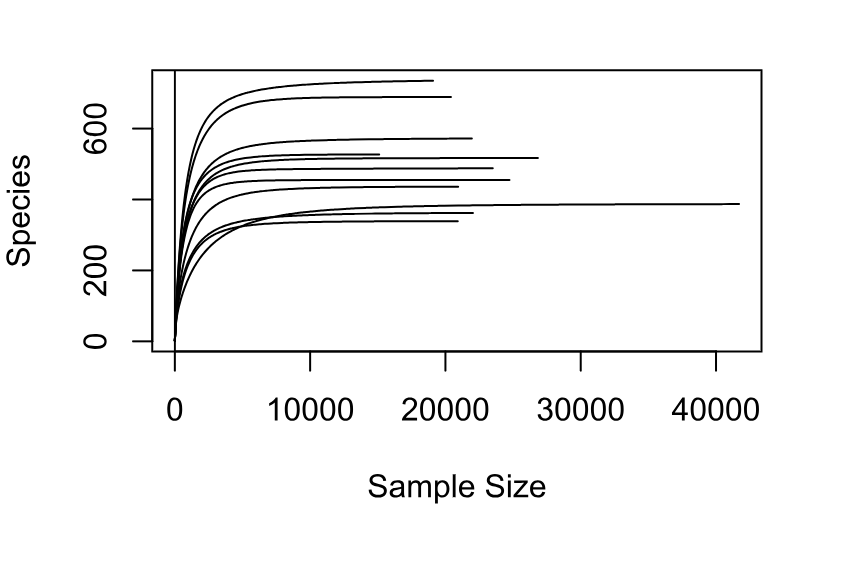


Supplementary Figure S1. Rarefaction curve of 16S reads


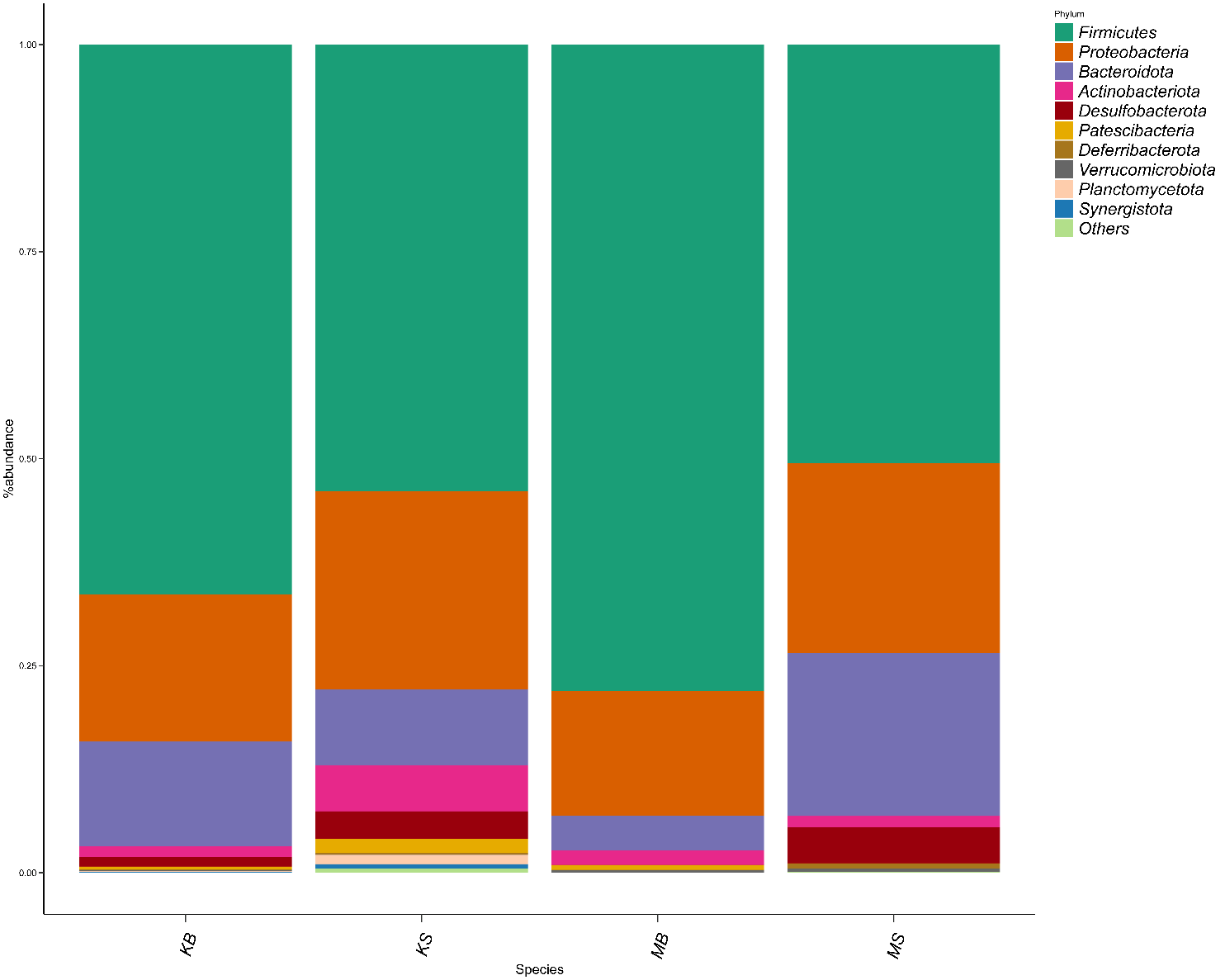


Supplementary Figure S2. Relative abundance of bacteria (16S) compositions at the phylum level. Abreviations; KS, KB, MS, MB are P. punctiger from Kakamega, T. jaspideus from Kakamega, P. punctiger from Mau and T. jaspideus from Mau, respectively.


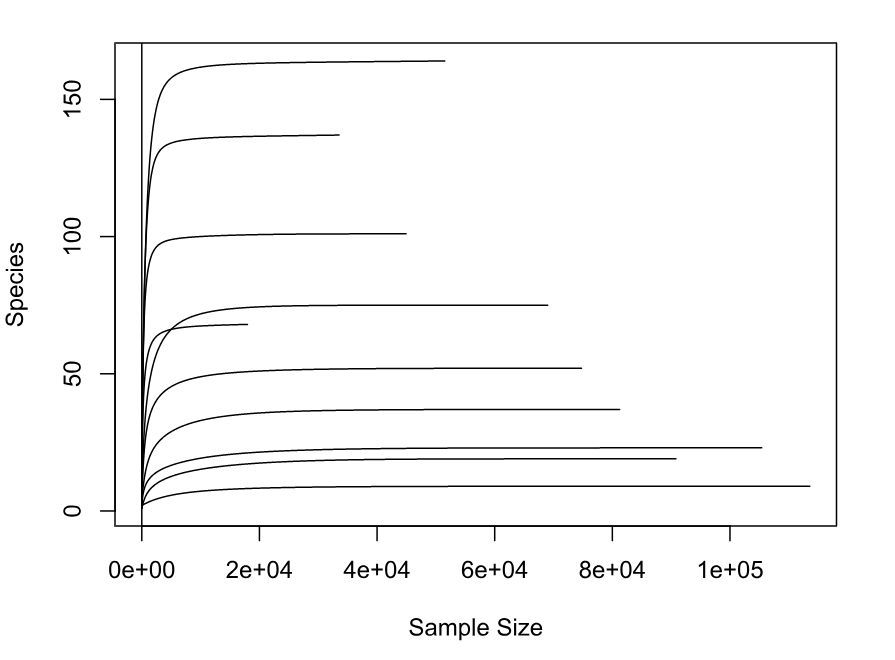


Supplementary Figure S3. Rarefaction curve of 18S reads


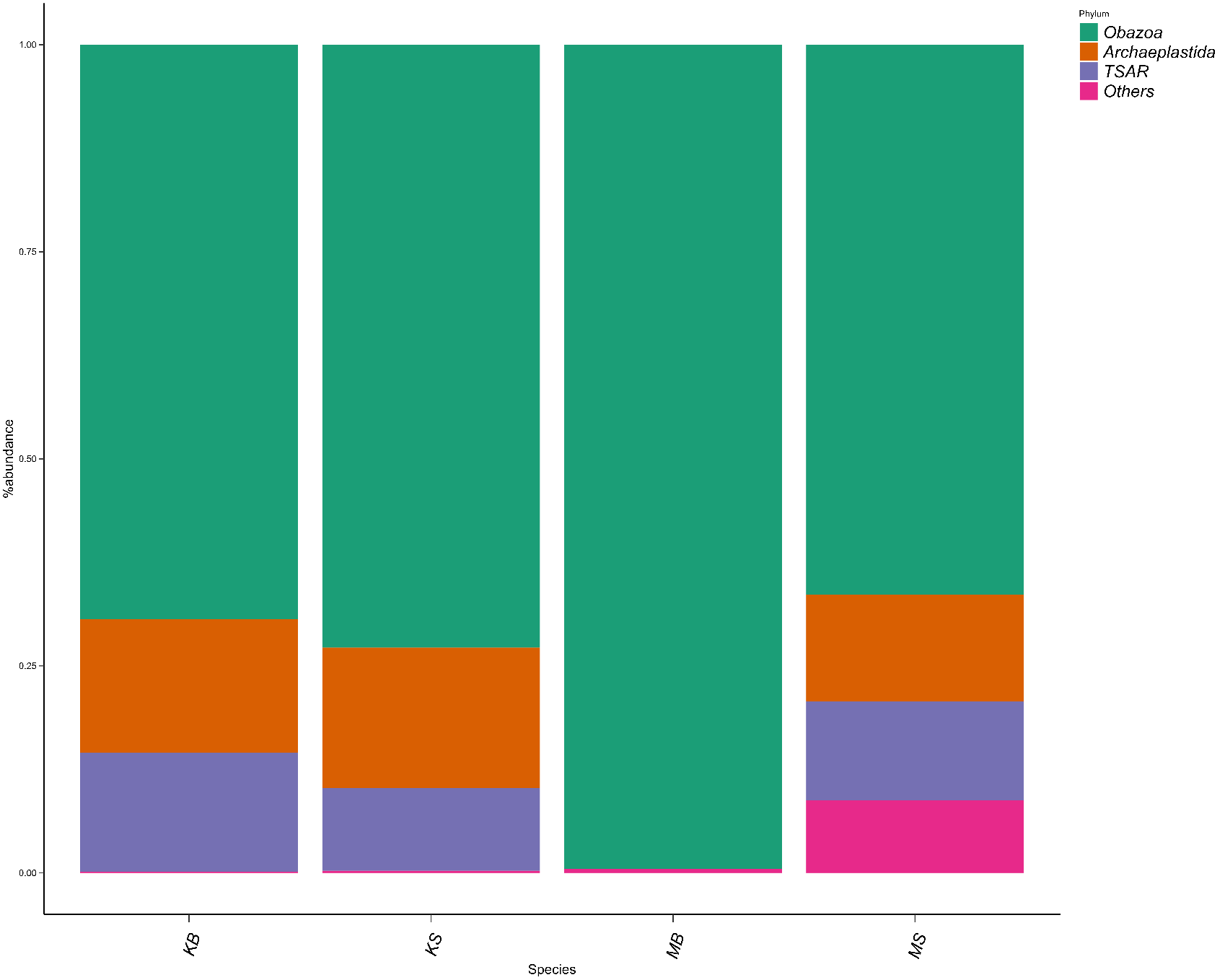


Supplementary Figure S4. Relative abundance of protists (18S) compositions at the phylum level. Abreviations; KS, KB, MS, MB are P. punctiger from Kakamega, T. jaspideus from Kakamega, P. punctiger from Mau and T. jaspideus from Mau, respectively.


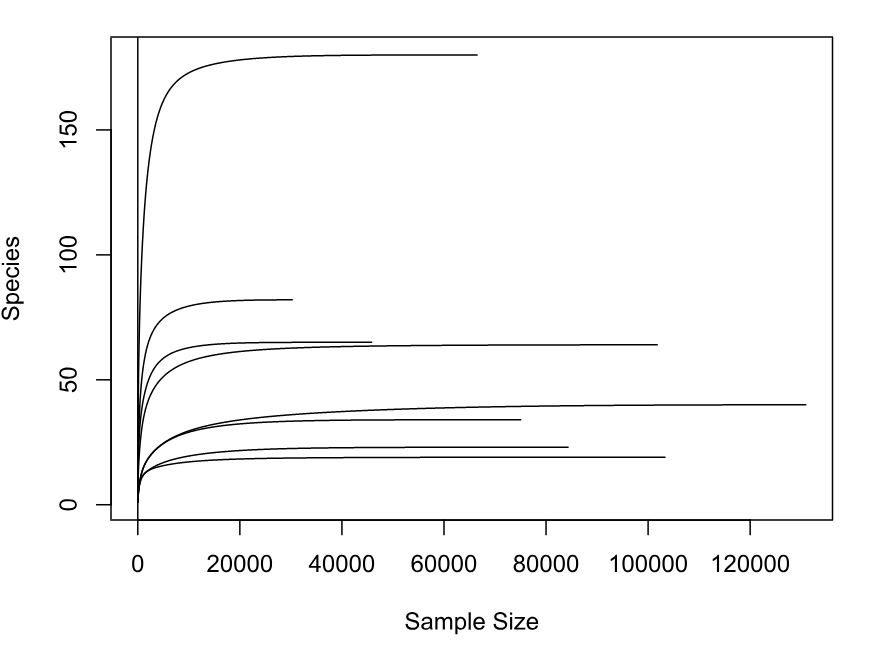


Supplementary Figure S5. Rarefaction curve of ITS reads


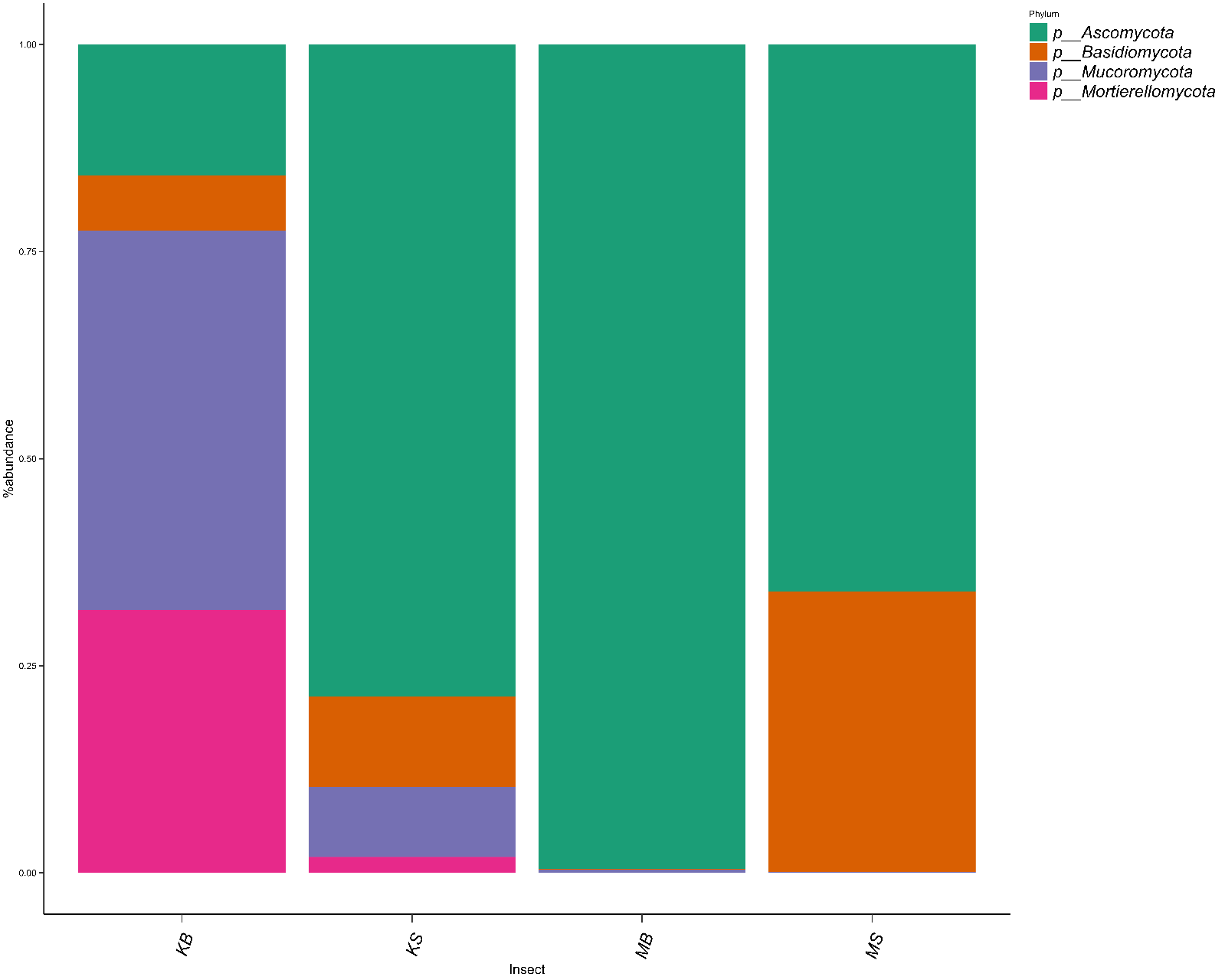


Supplementary Figure S6. Relative abundance of fungi (ITS) compositions at the phylum level. Abreviations; KS, KB, MS, MB are P. punctiger from Kakamega, T. jaspideus from Kakamega, P. punctiger from Mau and T. jaspideus from Mau, respectively.
